# A re-evaluation of LINE-1 ORF2 expression in LNCaP prostate cancer cells

**DOI:** 10.1186/s13100-019-0196-x

**Published:** 2019-12-29

**Authors:** Erica M. Briggs, Corrado Spadafora, Susan K. Logan

**Affiliations:** 1 Departments of Biochemistry and Molecular Pharmacology, New York University School of Medicine, Alexandria Center for Life Sciences, 450 East 29th Street, Room 323, New York, NY 10016 USA; 2 Departments of Urology, New York University School of Medicine, Alexandria Center for Life Sciences, 450 East 29th Street, Room 323, New York, NY 10016 USA; 3 Institute of Translational Pharmacology, National Research Council, Rome, Italy

**Keywords:** BCLAF1, ORF2, ORF2 antibody, LINE-1, Prostate cancer

## Abstract

**Background:**

We previously examined expression of Long Interspersed Element-1 (LINE-1) in a variety of prostate cancer cells including hormone-dependent LNCaP cells. These studies demonstrated expression and sub-cellular localization of LINE-1 proteins, ORF1p, with antibody 4H1, and ORF2p, with antibody chA1-L1.

**Results:**

Here we conduct immunoprecipitation/mass spectrometry analysis using chA1-L1 antibody against ORF2p in LNCaP cells. Our results indicate that antigens detected by the antibody include the transcriptional regulator BCLAF1. We show that chA1-L1 recognizes BCLAF1 using siRNA knockdown and overexpression of a tagged BCLAF1. We also show that chA1-L1 antibody recognizes ORF2p in HEK293 cells overexpressing LINE-1. Further, analysis of ORF2p (chA1-L1) and BCLAF1 foci using immunofluorescence in LNCaP cells showed significant colocalization.

**Conclusions:**

Overall, our findings indicate that chA1-L1 antibody recognizes both BCLAF1 and ORF2p but the majority of antigen recognized in LNCaP cells is BCLAF1.

## Findings

Long Interspersed Element-1 (LINE-1) is the only autonomous mobile element in the human genome. Its ability to mobilize, or retrotranspose, via an RNA intermediate generates additional copies of LINE-1 within the genome [1]. LINE-1 retrotransposition has led to the accumulation of LINE-1 sequences, occupying an estimated 17% of human DNA [2]. LINE-1 mRNA is bicistronic and codes for two proteins necessary for retrotransposition, ORF1p and ORF2p. ORF1p serves as a nucleic acid chaperone, while ORF2p contains endonuclease and reverse transcriptase enzymatic domains active in retrotransposition [3–5]. While LINE-1 mRNA has been detected in normal tissue, many mechanisms act to repress LINE-1 activity in somatic tissue to preserve genomic stability [6–9]. LINE-1 proteins are absent from most healthy somatic tissue, yet, LINE-1 protein expression and mobilization have been observed in a variety of cancers [10–12].

**Fig. 1:**
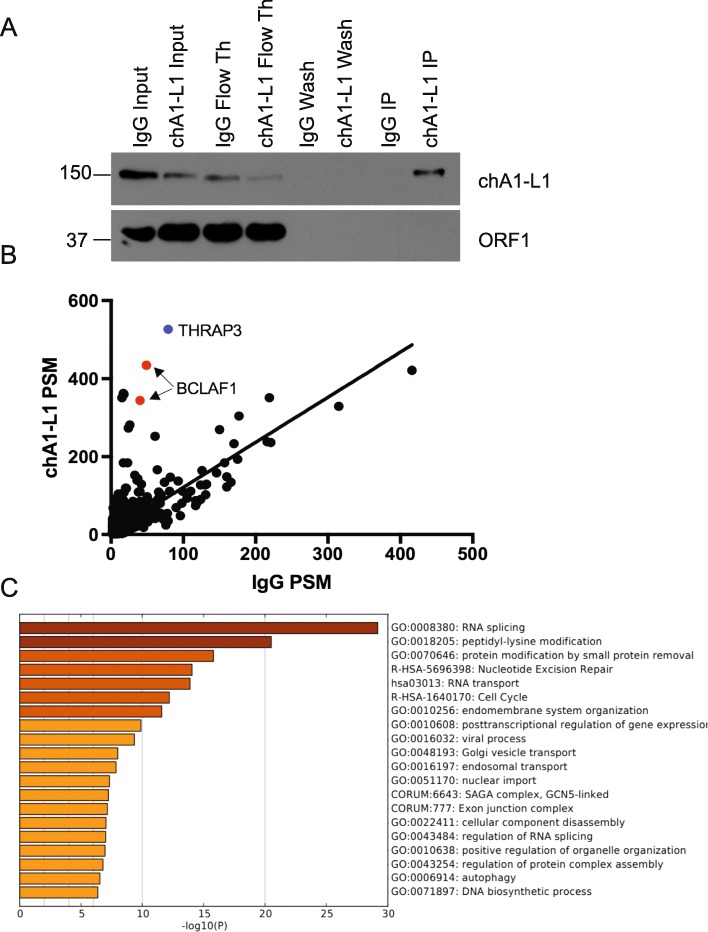
ORF2p/Immunoprecipitation Mass Spectroscopy. **a** Western blot of endogenous ORF2p immunoprecipitation from LNCaP nuclear extract using the chA1-L1 ORF2p antibody. Immunoprecipitated proteins were submitted for Mass Spectroscopy (MS) analysis. **b** Linear regression of chA1-L1 ORF2 IP peptide-spectrum match (PSM) and IgG IP peptide-spectrum match. Thrap3 (blue) and BCLAF1 (red) had two of the highest chA1-L1 PSM values. NUMA1 excluded due to high contamination in IgG control. **c** Metascape analysis of functional categories enriched in the chA1-L1 ORF2p IP [15]

In our previous publication, we characterized LINE-1 expression, retrotransposition frequency, and protein localization in prostate cancer cells [13]. Our findings demonstrated most prostate cancer cell lines expressed ORF1p, which was primarily localized in the cytoplasm. We utilized an ectopic retrotransposition assay to assess retrotransposition frequency in prostate cancer cells and found the highest rate of retrotransposition in LNCaP cells, followed by PC3 cells, 22Rv1 cells and very low levels in LAPC4 cells. Additionally, we utilized a newly available ORF2p antibody, chA1-L1, to characterize endogenous ORF2p expression in prostate cancer cells [14]. We demonstrated robust ORF2p expression in many prostate cancer cell lines, most of which was expressed as punctate foci localized in the nucleus. Since ORF2p endonuclease and reverse transcriptase domains have the potential to disrupt genomic stability, we hypothesized that prostate cancer cells were actively regulating ORF2p through prostate specific protein-protein interactions in the nucleus. To investigate this hypothesis, we immunoprecipitated endogenous ORF2p, using chA1-L1, from LNCaP nuclear lysates and conducted mass spectroscopy to identify ORF2p nuclear interactors. Surprisingly, our mass spectroscopy analysis did not identify any ORF2p peptides despite our western blot demonstrating a successful immunoprecipitation of ORF2p using chA1-L1 (Fig. 1a). Two of the top proteins identified in our mass spectroscopy were paralogous transcriptional regulators, BCLAF1 and THRAP3 (Fig. 1b). Initially, we suspected that our mass spectroscopy analysis was misidentifying ORF2 peptides due to sequence variability. To investigate these puzzling results further, we conducted a Metascape analysis of peptides unique to the ORF2p chA1-L1 immunoprecipitation [15]. Our Metascape analysis identified RNA processing and DNA damage repair pathways (RNA splicing, nucleocytoplasmic transport, nucleotide excision repair) as top pathways represented in the ORF2p chA1-L1 IP, consistent with ORF2p’s interaction with RNA and potential to induce DNA damage (Fig. 1c) [4, 16, 17]. Additionally, we speculated that ORF2 sequence variability might have contributed to the difficulty identifying ORF2p peptides in our mass spectroscopy analysis [18]. Due to these factors, we investigated the possible interaction between top proteins identified in our screen and ORF2p in LNCaP cells.

**Fig. 2:**
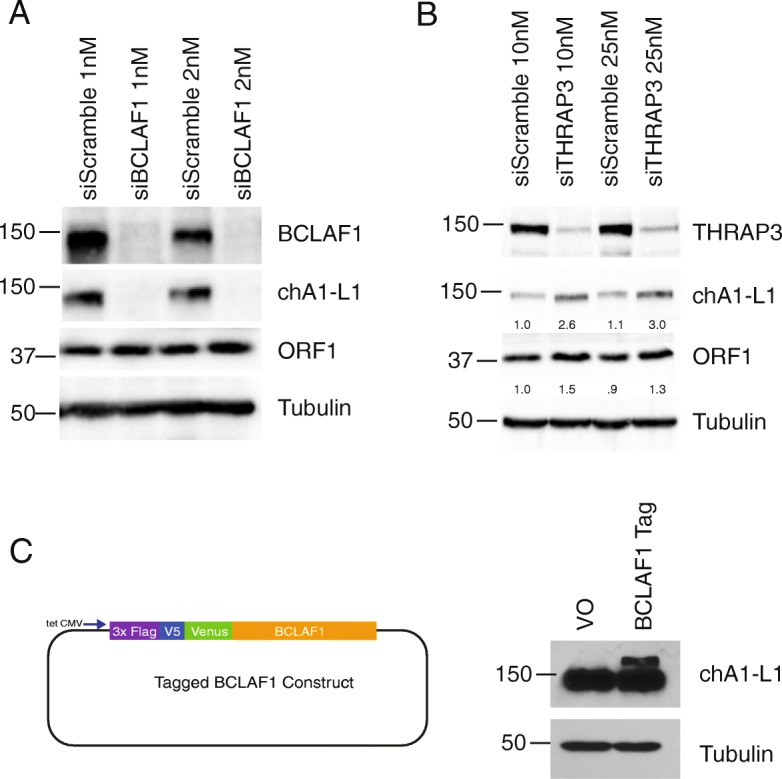
ORF2p chA1-L1 antibody cross-reacts with BCLAF1. LNCaP cells were treated with Scramble, BCLAF1 (**a**) or THRAP3 (**b**) siRNA for 72 h. Whole cell lysates were assayed via western blot and probed with chA1-L1 ORF2p antibody, BCLAF1 or THRAP3, ORF1p, and Tubulin (loading control). Intensity of ORF1 bands in **b** were normalized to tubulin levels. **c)** Left: BCLAF1 Tagged overexpression construct with N-terminus 3x flag, V5, and Venus tag (~ 35 kDa). Right: HEK293 cells were transfected with vector only (VO) or tagged BCLAF1 construct. Whole cell lysates were assayed by western blot and probed with ORF2p antibody chA1-L1, and Tubulin (loading control)

Since BCLAF1 and THRAP3 have both been shown to regulate RNA during DNA damage, we hypothesized that these proteins may play a role in regulating LINE-1 mRNA, thereby limiting LINE-1 protein expression and retrotransposition [19, 20]. To test whether BCLAF1 or THRAP3 had an effect on LINE-1 protein expression, we performed siRNA knockdown of BCLAF1 and THRAP3 in LNCaP cells (Fig. 2a and b). Upon THRAP3 knockdown, we observed a 1.4–1.5 fold increase in LINE-1 ORF1p and 2.6–2.7 fold increase in ORF2p (chA1-L1) expression. However, upon BCLAF1 knockdown, the band for ORF2p was significantly diminished while ORF1p protein levels remained relatively unchanged. Due to this discrepancy between ORF1p and ORF2p protein levels, we further investigated the relationship between BCLAF1 and ORF2p. Since ORF2p and BCLAF1 proteins have similar electrophoretic mobilities, with an apparent molecular weight of ~ 150 kDa, we explored the possibility that the ORF2p antibody, chA1-L1, also recognized BCLAF1 protein. To help distinguish overexpressed from endogenous BCLAF1, we created a BCLAF1 construct with a ~ 35 kDa tag on the N-terminus (Fig. 2c). Whole cell lysates transfected with vector only (VO) or the BCLAF1 tagged construct were run on a western blot and probed with ORF2p chA1-L1 antibody. The ORF2p chA1-L1 antibody detected the shifted band in the tagged BCLAF1 overexpression sample, as well as a lower band in both VO and overexpressed BCLAF1 consistent with the molecular weight of endogenous BCLAF1 protein. These experiments suggest that THRAP3 may play a role modulating LINE-1 mRNA or protein levels (Fig. 2b), however, further investigation is needed to confirm this effect. Additionally, our findings confirm that the ORF2p chA1-L1 antibody detects both overexpressed and endogenous BCLAF1 protein (Fig. 2a and c).

**Fig. 3:**
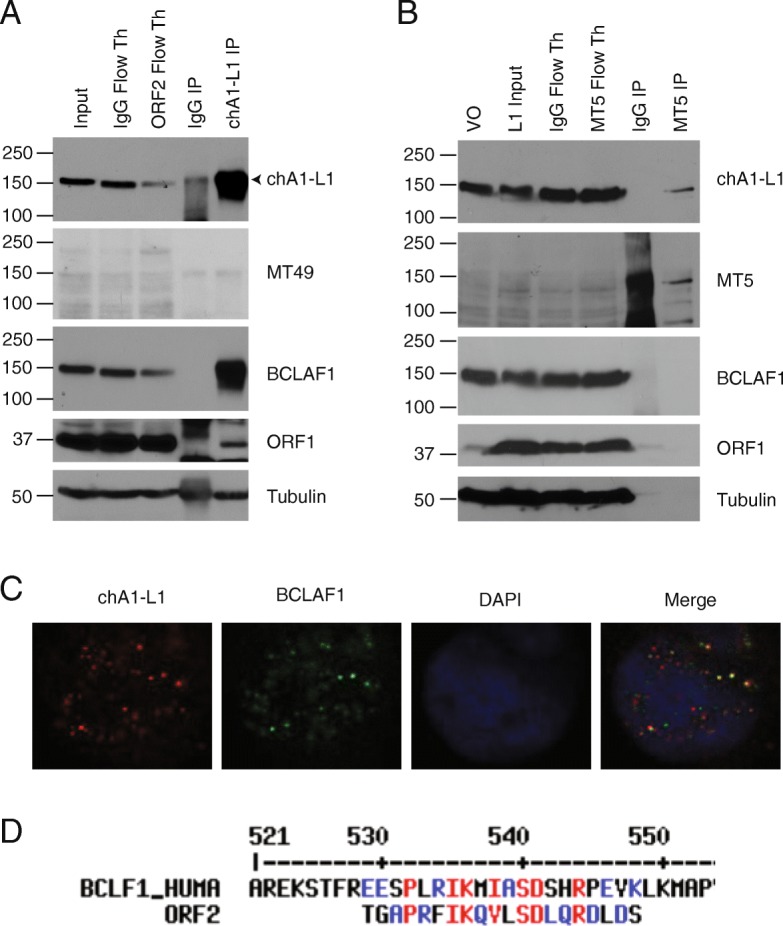
ORF2p Immunoprecipitation, immunofluorescence and protein alignment. **a** Whole cell LNCaP immunoprecipitation (IP) of ORF2p using chA1-L1 antibody. Input, flow through, and IPs were run on a western blot and probed for ORF2 (chA1-L1), ORF2 (MT49), BCLAF1, ORF1, and Tubulin (loading control). **b** HEK293 cells were transfected with LINE-1, a human CMV ORFeus recoded LINE-1 construct. ORF2p MT5 was used for immunoprecipitation of ORF2p in whole cell lysates overexpressing LINE-1. Vector only lysate (VO), and LINE1 (L1) Input, flow through, and IP (from LINE-1 overexpression lysate) were run on a western blot and probed with ORF2p MT5, ORF2p (chA1-L1), BCLAF1, and tubulin (loading control). **c** Confocal image of LNCaP cells that were probed for ORF2p using chA1-L1 (red), BCLAF1 (green) and nuclei stained with DAPI (Blue). **d** MultAlin protein alignment of human BCLAF1 and immunizing ORF2 chA1-L1 peptide

The question remained whether chA1-L1 solely detects BCLAF1 or whether it detects BCLAF1 and ORF2p. To address this, we performed two immunoprecipitations (IP) of ORF2p using the chA1-L1 antibody and two alternative ORF2p antibodies, MT5 and MT49, developed and generously provided by the laboratory of Kathleen Burns at John Hopkins University (see accompanying manuscript by Burns, LaCava and colleagues, 10.1101/744425). MT5 recognizes the reverse transcriptase domain of ORF2p (epitope QDIGVGKD), while MT49 recognizes the endonuclease domain (epitope DRSTRQ). We first used the ORF2p chA1-L1 antibody to immunoprecipitate endogenous ORF2p from LNCaP whole cell lysate (Fig. 3a, arrow). Western blot analysis showed a strong band at ~ 150 kDa when the IP was probed with the chA1-L1 antibody. A similar pattern was observed when we probed with a BCLAF1 antibody. Further, the BCLAF1 band was diminished in the ORF2p flow through lane suggesting once again that chA1-L1 recognizes BCLAF1. However, ORF1p also co-immunoprecipitated in the chA1-L1 IP, likely indicating that chA1-L1 is also pulling down ORF1p, which is well known to interact with ORF2p through RNA (Fig. 3a) [21]. Since the molecular weight of both BCLAF1 and ORF2p was ~ 150 kDa, we considered the possibility that BCLAF1 was co-immunoprecipitating with ORF2p. To address this, we probed with an alternative ORF2p antibody, MT49 (Burns laboratory, John Hopkins University). However, we did not detect any ORF2p in the input or immunoprecipitation using this antibody. We also tested MT5 antibody to immunoprecipitate overexpressed ORF2p in HEK293 cells (Fig. 3b). MT5 immunoprecipitated a band ~ 150 kDa, likely to be ORF2p. The chA1-L1 antibody also recognized this band, suggesting chA1-L1 also detects ORF2p. No BCLAF1 was detected in the MT5 ORF2p immunoprecipitation. Altogether, these results confirm that the chA1-L1 antibody recognizes both ORF2p and BCALF1 proteins.

In our previous publication, we also demonstrated ORF2p localized to punctate foci in the nucleus. Since we found chA1-L1 also detects BCLAF1, we wanted analyze these foci to see if they were also positive for BCLAF1. We conducted immunofluorescence for ORF2p chA1-L1 (red) and BCLAF1 (green) (Fig. 3c). We found significant co-localization of BCLAF1 and ORF2p foci, most likely representing chA1-L1 detection of BCLAF1. However, not all foci positive for ORF2p were positive for BCLAF1. While it is conceivable that these may be ORF2p foci, we have not eliminated the possibility that these are additional BCLAF1 foci that were not detected by the BCLAF1 antibody or that there are alternative off-target proteins detected by chA1-L1 antibody.

We next conducted protein sequence alignment using MultAlin software to assess whether the chA1-L1 immunizing peptide and BCLAF1 contained sequence similarities [22]. However, only weak sequence similarities were found (Fig. 3d). While we did not detect obvious sequence similarities, it is also possible that protein conformation or epitope spreading resulted in chA1-L1 recognition of BCLAF1.

Advancements in studying endogenous ORF2p have been hindered for many years due to low levels of expression and the lack of a reliable antibody, posing a significant challenge for scientists studying LINE-1. Additional mass spectroscopy, immunohistochemistry, and immunoprecipitation studies have recently confirmed the insurmountable challenge of detecting low levels of ORF2 in tumor samples and cell lines (see accompanying manuscript by Burns, LaCava and colleagues, 10.1101/744425). The development of ORF2p antibody chA1-L1 offered a glimpse into endogenous ORF2p expression, opening the door to understanding LINE-1 ORF2p regulation and activity in cancer cells. While this antibody was previously validated with LINE-1 overexpression and knockdown experiments, our findings show that in LNCaP prostate cancer cells, the chA1-L1 antibody also recognizes BCLAF1. Interestingly, BCLAF1 and its paralogue THRAP3 were both identified in a previous study to interact with LINE-1 proteins [21]. Both proteins were identified by IP mass spectroscopy of ORF1p and ORF2p, yet were not deemed significant by the investigators. It is possible that these proteins were identified in LINE-1 IPs because of their indirect interaction with RNA, or they may play an active role in LINE-1 regulation, a topic in need of further investigation. Additionally, it is unclear why chA1-L1 recognizes both ORF2p and BCLAF1. Yet, due to the similar molecular weight of ORF2p and BCLAF1, and widespread expression of BCLAF1 in other tissues (Human Protein Atlas [23]), it is problematic to use chA1-L1 to investigate ORF2p. Lastly, our findings stress the importance of independently validating all reagents.

## Methods

### Nuclear Immunoprecipitation

2 × 10^8^ LNCaP cells were fractionated into cytoplasmic and nuclear fractions as previously described [24]. The nuclear fraction was incubated with 400 μL Protein G Dynabeads (Invitrogen; 10003D) conjugated to 80 μg chA1-L1 overnight at 4 °C. Proteins were eluted by boiling samples in 2x SDS at 98 °C for 5 min. Immunoprecipitated proteins for chA1-L1 and IgG IPs were submitted for mass spectroscopy analysis.

### siRNA knockdown

siRNA knockdown was conducted using a reverse transfection with Lipofectamine RNAiMAX (Invitrogen; 13778030) according to the manufacturer’s protocol. 5 × 10^5^ LNCaP cells were treated with siRNA for 72 h (Ambion Silencer Select; BCLAF1- s61347; THRAP3 pool: s19360, s19361, s19359), and whole cell lysates were collected in RIPA buffer (50 mM Tris pH 8, 150 mM NaCl, 1% NP-40, 0.1% SDS, 10 mM EDTA, 10 μg/mL aprotonin and leuptin, 0.1 mM PMSF, and 0.1 mM Na_3_VO_4_). Protein concentration was quantified using a Bradford Assay.

### Western blot and immunofluorescence

Western blot and immunofluorescence were conducted as previously described [13]. Antibodies used include BCLAF1 (Bethyl; A300-608A), THRAP3 (Novus; NB100–40848), ORF1 (EMD Millipore MABC1152), and Tubulin (Covance MMS-489P). Immunofluorescent images were collected using a Laser scanning Zeiss 700 confocal microscope at 63x magnification and analyzed using ImageJ/Fiji [25].

### Immunoprecipitation

Cells were washed with cold PBS and lysed in 0.3 mL RIPA buffer (50 mM Tris pH 8, 150 mM NaCl, 1% NP-40, 0.1% SDS, 10 mM EDTA, 10 μg/mL aprotonin and leuptin, 0.1 mM PMSF, and 0.1 mM Na_3_VO_4_) per 10 cm plate. Protein concentration was measured using a Bradford assay. Cell lysates were incubated with 50 μL Protein G Dynabeads conjugated to MT5 (3.3 μg) or chA1-L1 (10μg) overnight at 4 °C. Proteins were eluted by boiling beads in 2x SDS loading buffer for 5 min at 98 °C.

### Plasmid construction

BCLAF1 tagged plasmid was built using gateway cloning (Invitrogen; 11791019, 11789013) with destination vector HuEV-A [26] and PCR amplified BCLAF1 (GenScript; OHu25921D) according to the manufacturer’s protocol. LINE-1 CMV ORFeus over expression construct was generously supplied by Dr. Jef Boeke [21].

### Cell culture

LNCaP cells (CRL-1740) were purchased from ATCC and maintained in RPMI 1640 with 10% FBS. HEK293 cells (CRL-1573) were purchased from ATCC and maintained in DMEM with 10% FBS. Cells were routinely checked for mycoplasma. For transfections, HEK293 cells were plated at 4.5 × 10^5^ in a 10 cm dish and transfected with Lipofectamine reagent according to the manufacturer’s protocol. Cells were lysed in RIPA as described above.

### Mass spectroscopy

The affinity purified proteins were reduced, alkylated, and loaded onto an SDS-PAGE gel to remove any detergents and LCMS incompatible reagents. The gel plugs were excised, destained, and subjected to proteolytic digestion with trypsin. The resulting peptides were extracted and desalted as previously described (Peled et al., 2017) and an aliquot of the peptides was analyzed with LCMS coupled to a ThermoFisher Orbitrap Fusion Lumos Mass Spectrometer operated in data dependent mode as previously described (Peled et al., 2017). The data was searched against a UniProt human database using Sequest within Proteome Discoverer.

## Data Availability

All data in this article is included in the published work.

## Abbreviations

BCLAF1: BCL2 Associated Transcription Factor 1
LINE-1: Long Interspersed Element 1
THRAP3: Thyroid Hormone Receptor Associated Protein 3
